# Methyl 2-[2-(2,6-dichloro­anilino)­phenyl]­acetate

**DOI:** 10.1107/S1600536808038336

**Published:** 2008-11-20

**Authors:** Rashid Saleem, Ghulam Shabir, Muhammad Hanif, Ghulam Qadeer, Wai-Yeung Wong

**Affiliations:** a Department of Chemistry, University of Engineering and Technology, Lahore, Pakistan; b Department of Chemistry, Quaid-I-Azam Univeristy, Islamabad 45320, Pakistan; c Department of Chemistry, Hong Kong Baptist University, Waterloo Road, Kowloon Tong, Hong Kong, People’s Republic of China

## Abstract

In the title compound, C_15_H_13_Cl_2_NO_2_, the dihedral angle between the aromatic rings is 63.80 (12)°. The conformation may be stabilized by a weak N—H⋯O hydrogen bond. In the crystal structure, a short C—Cl⋯π interaction occurs, with a Cl⋯π separation of 3.5706 (13) Å.

## Related literature

For general background, see: Hashem *et al.* (2007 ▶); Husain *et al.* (2005 ▶). For bond-length data, see: Allen *et al.* (1987 ▶).
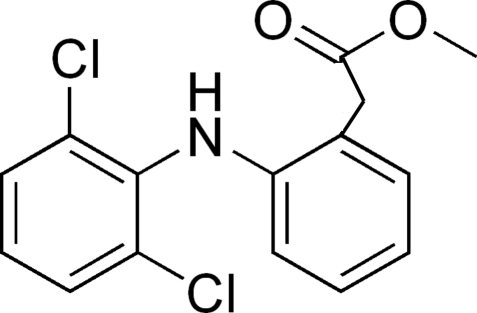


## Experimental

### 

#### Crystal data

C_15_H_13_Cl_2_NO_2_
*M* *_r_* = 310.16
Monoclinic, 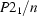

*a* = 4.9319 (4) Å
*b* = 20.0288 (14) Å
*c* = 14.5542 (10) Å
β = 97.711 (1)°
*V* = 1424.66 (18) Å^3^
*Z* = 4
Mo *K*α radiation
μ = 0.46 mm^−1^
*T* = 173 (2) K
0.38 × 0.24 × 0.20 mm

#### Data collection

Bruker SMART APEXII CCD diffractometer
Absorption correction: multi-scan (*SADABS*; Bruker, 2005 ▶) *T*_min_ = 0.850, *T*_max_ = 1.000 (expected range = 0.776–0.913)
8526 measured reflections
3423 independent reflections
2777 reflections with *I* > 2σ(*I*)
*R*_int_ = 0.020

#### Refinement

*R*[*F* ^2^ > 2σ(*F* ^2^)] = 0.052
*wR*(*F* ^2^) = 0.171
*S* = 1.04
3423 reflections
181 parameters
H-atom parameters constrained
Δρ_max_ = 0.62 e Å^−3^
Δρ_min_ = −0.52 e Å^−3^

### 

Data collection: *APEX2* (Bruker, 2005 ▶); cell refinement: *SAINT* (Bruker, 2007 ▶); data reduction: *SAINT*; program(s) used to solve structure: *SHELXS97* (Sheldrick, 2008 ▶); program(s) used to refine structure: *SHELXL97* (Sheldrick, 2008 ▶); molecular graphics: *SHELXTL* (Sheldrick, 2008 ▶); software used to prepare material for publication: *SHELXTL*, *PARST* (Nardelli, 1995 ▶) and *PLATON* (Spek, 2003 ▶).

## Supplementary Material

Crystal structure: contains datablocks I, global. DOI: 10.1107/S1600536808038336/pv2122sup1.cif

Structure factors: contains datablocks I. DOI: 10.1107/S1600536808038336/pv2122Isup2.hkl

Additional supplementary materials:  crystallographic information; 3D view; checkCIF report

## Figures and Tables

**Table 1:** Hydrogen-bond geometry (Å, °)

*D*—H⋯*A*	*D*—H	H⋯*A*	*D*⋯*A*	*D*—H⋯*A*
N1—H1*A*⋯O1	0.88	2.64	3.152 (2)	118

**Fig. 1.**
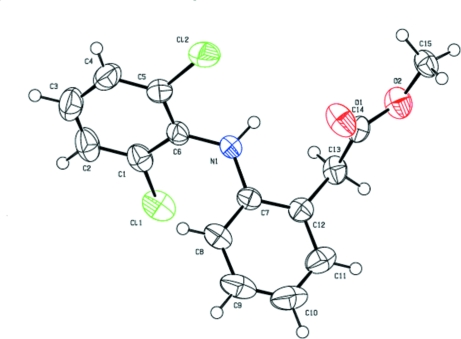
The molecular structure of the title compound, with the atom-numbering scheme; thermal ellipsoids have been plotted at 50% probability level.

**Fig. 2.**
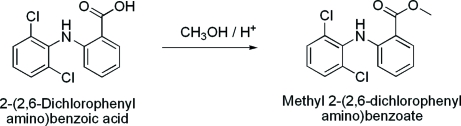
The formation of the title compound.

## supplementary crystallographic information

### Comment

Esters are important intermediates in heterocyclic chemistry and have been used for the synthesis of various biologically active five-membered heterocyles such as butenolides, pyrrolones (Husain *et al.*, 2005), oxadiazoles and triazoles (Hashem *et al.*, 2007). In view of the versatility of these compounds, we have synthesized the title compound and report herein its crystal structure.

In the title compound (Fig. 1), the bond lengths and angles are within normal ranges (Allen *et al.*, 1987). The planar ester group (O1/O2/C13/C14/C15) is oriented with respect to the plane of the benzene ring (C7–C12) at an angle 41.33 (2)°. There is a short intramolecular N—H···O hydrogen bond (Table 1) and a π-ring interaction of the type C—Cl···Cg with Cl1···Cg1 (centroid of C1–C6 ring) perpendicular distance 3.5706 (13) Å.

### Experimental

A mixture of 2-(2,6-dichlorophenylamino)benzoic acid (2.81 g, 10 mmol) and absolute methanol (50 ml) in the presence of a few drops of sulfuric acid was refluxed for 5 h. The excess of the solvent was removed by distillation. The solid residue was filltered off, washed with water and recystallized from ethanol (30%) to give the title compound. Suitable single crystals of the title compound were obtained by slow evaporation of an ethanol solution at room temperature. (Yield, 88%; m.p. 331–332 K)

### Refinement

H atoms were positioned geometrically, with O—H = 0.82 Å and C—H = 0.93, 0.97 and 0.96 Å for aryl, methylene and methyl H, respectively, and constrained to ride on their parent atoms with *U*_iso_(H) = 1.5*U*_eq_(methyl C) and 1.2*U*_eq_(aryl and methylene C and O)

### Crystal data

**Table:**

C_15_H_13_Cl_2_NO_2_	*F*_000_ = 640
*M**_r_* = 310.16	*D*_x_ = 1.446 Mg m^−^^3^
Monoclinic, *P*2_1_/*n*	Melting point: 331 K
Hall symbol: -P 2yn	Mo *K*α radiation λ = 0.71073 Å
*a* = 4.9319 (4) Å	Cell parameters from 9949 reflections
*b* = 20.0288 (14) Å	θ = 2.4–28.3º
*c* = 14.5542 (10) Å	µ = 0.46 mm^−^^1^
β = 97.711 (1)º	*T* = 173 (2) K
*V* = 1424.66 (18) Å^3^	Block, pale yellow
*Z* = 4	0.38 × 0.24 × 0.20 mm

### Data collection

**Table:**

Bruker SMART APEXII CCD diffractometer	3423 independent reflections
Radiation source: fine-focus sealed tube	2777 reflections with *I* > 2σ(*I*)
Monochromator: graphite	*R*_int_ = 0.020
*T* = 173(2) K	θ_max_ = 28.3º
ω and φ scans	θ_min_ = 2.5º
Absorption correction: multi-scan(SADABS; Bruker, 2005)	*h* = −6→6
*T*_min_ = 0.850, *T*_max_ = 1.000	*k* = −26→26
8526 measured reflections	*l* = −19→15

### Refinement

**Table:**

Refinement on *F*^2^	Secondary atom site location: difference Fourier map
Least-squares matrix: full	Hydrogen site location: inferred from neighbouring sites
*R*[*F*^2^ > 2σ(*F*^2^)] = 0.052	H-atom parameters constrained
*wR*(*F*^2^) = 0.171	*w* = 1/[σ^2^(*F*_o_^2^) + (0.1042*P*)^2^ + 0.5254*P*] where *P* = (*F*_o_^2^ + 2*F*_c_^2^)/3
*S* = 1.04	(Δ/σ)_max_ < 0.001
3423 reflections	Δρ_max_ = 0.62 e Å^−^^3^
181 parameters	Δρ_min_ = −0.52 e Å^−^^3^
Primary atom site location: structure-invariant direct methods	Extinction correction: none

### Special details

**Table:**

Geometry. All e.s.d.'s (except the e.s.d. in the dihedral angle between two l.s. planes) are estimated using the full covariance matrix. The cell e.s.d.'s are taken into account individually in the estimation of e.s.d.'s in distances, angles and torsion angles; correlations between e.s.d.'s in cell parameters are only used when they are defined by crystal symmetry. An approximate (isotropic) treatment of cell e.s.d.'s is used for estimating e.s.d.'s involving l.s. planes.
Refinement. Refinement of *F*^2^ against ALL reflections. The weighted *R*-factor *wR* and goodness of fit *S* are based on *F*^2^, conventional *R*-factors *R* are based on *F*, with *F* set to zero for negative *F*^2^. The threshold expression of *F*^2^ > σ(*F*^2^) is used only for calculating *R*-factors(gt) *etc*. and is not relevant to the choice of reflections for refinement. *R*-factors based on *F*^2^ are statistically about twice as large as those based on *F*, and *R*- factors based on ALL data will be even larger.

### Fractional atomic coordinates and isotropic or equivalent isotropic displacement parameters (Å^2^)

**Table:**

	*x*	*y*	*z*	*U*_iso_*/*U*_eq_	
C1	0.8063 (4)	0.18670 (11)	0.42183 (15)	0.0459 (5)	
C2	0.9559 (6)	0.23352 (14)	0.47685 (18)	0.0592 (7)	
H2A	0.9401	0.2361	0.5411	0.071*	
C3	1.1298 (6)	0.27691 (13)	0.4384 (2)	0.0646 (7)	
H3A	1.2365	0.3084	0.4764	0.077*	
C4	1.1462 (6)	0.27396 (11)	0.3451 (2)	0.0572 (6)	
H4A	1.2586	0.3046	0.3178	0.069*	
C5	0.9985 (5)	0.22618 (10)	0.29100 (16)	0.0454 (5)	
C6	0.8295 (4)	0.17922 (9)	0.32730 (13)	0.0386 (4)	
C7	0.6955 (4)	0.06202 (9)	0.29122 (13)	0.0360 (4)	
C8	0.8988 (5)	0.03457 (12)	0.35515 (15)	0.0468 (5)	
H8A	1.0348	0.0624	0.3879	0.056*	
C9	0.9027 (6)	−0.03407 (14)	0.37114 (18)	0.0614 (7)	
H9A	1.0410	−0.0529	0.4153	0.074*	
C10	0.7088 (7)	−0.07451 (13)	0.3237 (2)	0.0676 (8)	
H10A	0.7142	−0.1213	0.3347	0.081*	
C11	0.5040 (5)	−0.04753 (12)	0.25957 (19)	0.0549 (6)	
H11A	0.3696	−0.0759	0.2271	0.066*	
C12	0.4948 (4)	0.02126 (10)	0.24258 (14)	0.0388 (4)	
C13	0.2660 (4)	0.04998 (12)	0.17557 (15)	0.0434 (5)	
H13A	0.1975	0.0907	0.2033	0.052*	
H13B	0.1140	0.0173	0.1670	0.052*	
C14	0.3438 (4)	0.06753 (10)	0.08154 (14)	0.0374 (4)	
C15	0.1597 (4)	0.09199 (12)	−0.07671 (14)	0.0433 (5)	
H15A	−0.0226	0.0958	−0.1125	0.065*	
H15B	0.2626	0.0566	−0.1032	0.065*	
H15C	0.2570	0.1345	−0.0789	0.065*	
Cl1	0.57692 (13)	0.13766 (4)	0.47192 (4)	0.0602 (2)	
Cl2	1.01835 (17)	0.22543 (3)	0.17271 (4)	0.0654 (2)	
N1	0.6851 (4)	0.13126 (8)	0.27114 (12)	0.0425 (4)	
H1A	0.5820	0.1447	0.2205	0.051*	
O1	0.5844 (3)	0.07442 (10)	0.06748 (12)	0.0591 (5)	
O2	0.1341 (3)	0.07623 (9)	0.01586 (12)	0.0550 (4)	

### Atomic displacement parameters (Å^2^)

**Table:**

	*U*^11^	*U*^22^	*U*^33^	*U*^12^	*U*^13^	*U*^23^
C1	0.0417 (11)	0.0525 (12)	0.0422 (11)	0.0138 (9)	0.0005 (8)	−0.0030 (9)
C2	0.0595 (15)	0.0663 (15)	0.0479 (12)	0.0187 (12)	−0.0069 (10)	−0.0198 (11)
C3	0.0656 (16)	0.0500 (13)	0.0718 (17)	0.0071 (11)	−0.0138 (13)	−0.0208 (12)
C4	0.0564 (14)	0.0363 (10)	0.0754 (17)	−0.0006 (9)	−0.0040 (12)	−0.0007 (10)
C5	0.0512 (12)	0.0358 (9)	0.0473 (11)	0.0055 (8)	−0.0006 (9)	0.0021 (8)
C6	0.0373 (10)	0.0373 (9)	0.0385 (10)	0.0064 (7)	−0.0048 (7)	−0.0008 (7)
C7	0.0370 (9)	0.0388 (9)	0.0331 (9)	0.0030 (7)	0.0075 (7)	0.0035 (7)
C8	0.0460 (11)	0.0554 (12)	0.0386 (10)	0.0100 (9)	0.0045 (8)	0.0072 (9)
C9	0.0735 (17)	0.0627 (15)	0.0506 (13)	0.0289 (13)	0.0176 (12)	0.0193 (11)
C10	0.097 (2)	0.0419 (12)	0.0690 (17)	0.0124 (13)	0.0313 (16)	0.0124 (11)
C11	0.0661 (15)	0.0420 (11)	0.0606 (14)	−0.0056 (10)	0.0230 (12)	0.0000 (10)
C12	0.0384 (10)	0.0412 (10)	0.0385 (10)	−0.0019 (7)	0.0111 (8)	0.0003 (7)
C13	0.0311 (9)	0.0565 (12)	0.0430 (11)	−0.0068 (8)	0.0073 (8)	−0.0036 (9)
C14	0.0286 (9)	0.0407 (9)	0.0436 (10)	−0.0040 (7)	0.0075 (7)	−0.0060 (8)
C15	0.0329 (10)	0.0601 (12)	0.0359 (10)	−0.0045 (8)	0.0005 (7)	0.0023 (8)
Cl1	0.0537 (4)	0.0771 (4)	0.0521 (4)	0.0141 (3)	0.0159 (3)	0.0074 (3)
Cl2	0.0911 (5)	0.0551 (4)	0.0509 (4)	−0.0072 (3)	0.0123 (3)	0.0118 (2)
N1	0.0489 (10)	0.0383 (8)	0.0363 (8)	−0.0016 (7)	−0.0084 (7)	0.0037 (6)
O1	0.0273 (7)	0.1015 (14)	0.0486 (9)	−0.0057 (7)	0.0057 (6)	0.0102 (9)
O2	0.0413 (8)	0.0703 (11)	0.0532 (10)	−0.0023 (7)	0.0060 (7)	−0.0026 (8)

### Geometric parameters (Å, °)

**Table:**

C1—C2	1.381 (3)	C9—C10	1.367 (5)
C1—C6	1.404 (3)	C9—H9A	0.9500
C1—Cl1	1.731 (3)	C10—C11	1.390 (4)
C2—C3	1.390 (4)	C10—H10A	0.9500
C2—H2A	0.9500	C11—C12	1.399 (3)
C3—C4	1.371 (4)	C11—H11A	0.9500
C3—H3A	0.9500	C12—C13	1.503 (3)
C4—C5	1.383 (3)	C13—C14	1.511 (3)
C4—H4A	0.9500	C13—H13A	0.9900
C5—C6	1.406 (3)	C13—H13B	0.9900
C5—Cl2	1.738 (2)	C14—O1	1.239 (2)
C6—N1	1.393 (3)	C14—O2	1.322 (3)
C7—C8	1.387 (3)	C15—O2	1.406 (3)
C7—C12	1.400 (3)	C15—H15A	0.9800
C7—N1	1.417 (2)	C15—H15B	0.9800
C8—C9	1.394 (4)	C15—H15C	0.9800
C8—H8A	0.9500	N1—H1A	0.8800
			
C2—C1—C6	122.2 (2)	C9—C10—H10A	119.8
C2—C1—Cl1	118.01 (19)	C11—C10—H10A	119.8
C6—C1—Cl1	119.72 (17)	C10—C11—C12	120.3 (2)
C1—C2—C3	120.1 (2)	C10—C11—H11A	119.8
C1—C2—H2A	120.0	C12—C11—H11A	119.8
C3—C2—H2A	120.0	C11—C12—C7	118.7 (2)
C4—C3—C2	119.5 (2)	C11—C12—C13	119.7 (2)
C4—C3—H3A	120.2	C7—C12—C13	121.54 (18)
C2—C3—H3A	120.2	C12—C13—C14	114.64 (16)
C3—C4—C5	119.8 (3)	C12—C13—H13A	108.6
C3—C4—H4A	120.1	C14—C13—H13A	108.6
C5—C4—H4A	120.1	C12—C13—H13B	108.6
C4—C5—C6	122.8 (2)	C14—C13—H13B	108.6
C4—C5—Cl2	118.4 (2)	H13A—C13—H13B	107.6
C6—C5—Cl2	118.79 (16)	O1—C14—O2	122.7 (2)
N1—C6—C1	123.1 (2)	O1—C14—C13	122.73 (18)
N1—C6—C5	121.52 (19)	O2—C14—C13	114.61 (16)
C1—C6—C5	115.29 (19)	O2—C15—H15A	109.5
C8—C7—C12	120.44 (19)	O2—C15—H15B	109.5
C8—C7—N1	121.95 (19)	H15A—C15—H15B	109.5
C12—C7—N1	117.59 (17)	O2—C15—H15C	109.5
C7—C8—C9	119.7 (2)	H15A—C15—H15C	109.5
C7—C8—H8A	120.1	H15B—C15—H15C	109.5
C9—C8—H8A	120.1	C6—N1—C7	123.53 (16)
C10—C9—C8	120.5 (2)	C6—N1—H1A	118.2
C10—C9—H9A	119.8	C7—N1—H1A	118.2
C8—C9—H9A	119.8	C14—O2—C15	124.07 (17)
C9—C10—C11	120.3 (2)		

### Hydrogen-bond geometry (Å, °)

**Table:**

*D*—H···*A*	*D*—H	H···*A*	*D*···*A*	*D*—H···*A*
N1—H1A···O1	0.88	2.64	3.152 (2)	118
